# Dr. Arthur Victor Conley

**Published:** 1895-09

**Authors:** 


Dr. Arthur Victor Conley, house-physician at the Buffalo Hospital of the Sisters of Charity, died of typhoid fever at that institution, August 23, 1895, after an illness of three weeks' duration, aged twenty-nine years. He graduated in medicine May 16, 1895, from Niagara University Medical College, standing at the head of his class. He was a young man of great promise and his early death is greatly to be lamented.

